# Supplementary material for: Genetic Diversity of Human Respiratory Syncytial Virus during COVID-19 Pandemic in Yaoundé, Cameroon, 2020–2021
Source: Microorganisms. 2024 May 8;12(5):952. doi: 10.3390/microorganisms12050952 (PMC11123827; doi:10.3390/microorganisms12050952)
Supplement: Supplementary file 1 [file microorganisms-12-00952-s001.zip › Table S1.pdf]

S1 table. Reference sequences used to construct Fig1

Number	Genbank number	Year	Genotype	Origin
1	KU316165	1987	GA1	USA
2	Z33427	1990	GA1	Uruguay
3	KJ723483	1984	GA2.1	USA
4	MG642070	1986	GA2.1	USA
5	JF920062	1998	GA2.2	USA
6	JX069801	1998	GA2.2	USA
7	KP119748	2012	GA2.3.0	HongKong
8	KU950573	2006	GA2.3.0	USA
9	EU025215	2005	GA2.3.1	Italy
10	KJ627305	2007	GA2.3.1	Peru
11	KT765718	2006	GA2.3.2a	Kenya
12	KT765726	2005	GA2.3.2a	Kenya
13	KJ627284	2010	GA2.3.2b	Peru
14	KJ627336	2008	GA2.3.2b	Peru
15	KJ627349	2009	GA2.3.3	Peru
16	KP317953	2012	GA2.3.3	Kenya
17	KJ672482	2012	GA2.3.4	USA
18	KU950667	2012	GA2.3.4	USA
19	JN257693	2010	GA2.3.5	Canada
20	KJ672470	2012	GA2.3.5	USA
21	KJ672467	2013	GA2.3.5	USA
22	KU950531	2013	GA2.3.5	USA
23	KU950540	2013	GA2.3.5	USA
24	KU950550	2012	GA2.3.5	USA
25	KX765917	2014	GA2.3.5	New Zealand
26	KX765932	2015	GA2.3.5	New Zealand
27	KY654514	2013	GA2.3.5	Philippines
28	KX894807	2013	GA2.3.5	USA
29	KY654518	2013	GA2.3.5	Philippines
30	KY883567	2015	GA2.3.5	Argentina
31	KT326808	2015	GA2.3.6a	Spain
32	KU350793	2012	GA2.3.6a	Argentina
33	KX453353	2013	GA2.3.6b	Kenya

34	KX453385	2014	GA2.3.6b	Kenya
35	HQ731716	1981	GA2	England
36	MG642063	1982	GA2	USA
37	DQ985132	1997	GA3.0.0	Belgium
38	KP258715	1988	GA3.0.0	USA
39	KP258699	1985	GA3.0.1	USA
40	MG642031	1982	GA3.0.1	USA
41	KJ723465	1992	GA3.0.2	USA
42	KP258701	1994	GA3.0.2	USA
43	JX069802	1998	GA3.0.3a	USA
44	KM360090	2001	GA3.0.3a	USA
45	AY910792	2000	GA3.0.3b	Argentina
46	JX513302	2002	GA3.0.3b	Brazil
47	KF826854	2009	GA3.0.4a	Italy
48	DQ985748	2006	GA3.0.4a	Turkey
49	KF826850	2008	GA3.0.4b	USA
50	KF973333	2002	GA3.0.4b	USA
51	KC297367	2012	GA3.0.5b	China
52	KF826832	2009	GA3.0.5b	Italy
