# Supplementary material for: Genetic Diversity of Human Respiratory Syncytial Virus during COVID-19 Pandemic in Yaoundé, Cameroon, 2020–2021
Source: Microorganisms. 2024 May 8;12(5):952. doi: 10.3390/microorganisms12050952 (PMC11123827; doi:10.3390/microorganisms12050952)
Supplement: Supplementary file 1 [file microorganisms-12-00952-s001.zip › Table S2.pdf]

S2 table. Reference sequences used to construct Fig2

Number	Genbank number	Year	Genotype	Origin
1	AY353550	1977	GB1	USA
2	KP258736	1982	GB1	USA
3	HQ731708	1985	GB2	England
4	M73540	1980	GB2	USA
5	AY333361	1990	GB3	Uruguay
6	JX198166	1993	GB3	USA
7	AF193331	1991	GB4	South Korea
8	JX198160	1993	GB4	USA
9	KF826853	2008	GB5.0.0	Germany
10	KP317923	2012	GB5.0.0	Kenya
11	AY751111	2003	GB5.0.1	Belgium
12	KF826829	2005	GB5.0.1	Mexico
13	HQ731688	2006	GB5.0.2	Scotland
14	KC297492	2009	GB5.0.2	China
15	JN032117	2006	GB5.0.3	USA
16	KF246627	2010	GB5.0.3	India
17	KM402687	2013	GB5.0.4a	Spain
18	KY249677	2012	GB5.0.4a	England
19	JX576746	2008	GB5.0.4b	Netherlands
20	KJ627285	2011	GB5.0.4b	Peru
21	KJ939928	2010	GB5.0.4c	Vietnam
23	KP862515	2012	GB5.0.4c	Kenya
24	KX765906	2015	GB5.0.5a	New Zealand
25	KY249683	2016	GB5.0.5a	England
26	MG773268	2016	GB5.0.5a	Argentina
27	MG839547	2016	GB5.0.5a	Argentina
28	KX775765	2014	GB5.0.5c	Kenya
28	KX775767	2013	GB5.0.5c	Kenya
29	JF704213	1998	GB6	South Africa
30	KP862078	2003	GB6	Kenya
31	KP862095	2004	GB6	Kenya
